# Comparative performance of predictive nomograms for pathological upstaging in clinical T1 renal masses: an independent external validation

**DOI:** 10.1007/s11255-026-05232-y

**Published:** 2026-06-09

**Authors:** Yanay Shaked, Boris Kaptzan, Ron Marom, Michael Vainrib, Amos Neheman, Omri Schwarztuch Gildor

**Affiliations:** 1 Department of Urology, Meir Medical Center, 59 Tchernichovsky St, 4428164 Kfar Saba, Israel; 2 Faculty of Medical and Health Sciences, Tel Aviv University, Tel Aviv, Israel

**Keywords:** Carcinoma, Renal cell, Nomograms, Validation studies as topic, Decision support techniques, Calibration

## Abstract

**Background:**

Preoperative identification of pathological upstaging (≥ pT3a) in clinical T1 (cT1) renal masses is critical for surgical planning. We externally validated three established predictive models, Barra, Nocera, and Mei, to assess their discrimination, calibration, and clinical utility in a contemporary cohort.

**Patients and methods:**

We retrospectively reviewed patients undergoing surgery for cT1 renal masses at our institution. The primary endpoint was pathological upstaging to pT3a. Model performance was evaluated using the Area Under the Curve (AUC) for discrimination and the Brier Score for overall accuracy. Calibration was assessed using parametric and nonparametric Loess-smoothed curves. Clinical utility was quantified using Standardized Decision Curve Analysis.

**Results:**

The institutional upstaging rate was 17.1% (19/111), and clear cell histology predominated (77.5%). Nocera (AUC = 0.736) and Barra (AUC = 0.733) had higher point estimates of discriminative ability than Mei (AUC = 0.677), but the differences were not significant. All models descriptively overestimated absolute risk. However, Mei demonstrated the highest absolute calibration stability, whereas the Nocera model showed significant global unreliability (U = 0.052, *p* = 0.021). The Barra model achieved the lowest overall error (Brier Score = 0.122) and the highest clinical net benefit within the decisive 8%–18% decision window, although the overall accuracy differences were not statistically significant.

**Conclusion:**

All nomograms are useful risk-stratification adjuncts, but wide confidence intervals and low event counts mean these findings are exploratory. No single model is definitively superior. Nocera and Barra are effective for relative risk-ranking, while Mei provides superior absolute precision for patient counseling.

## Introduction

The management of clinical T1 (cT1) renal masses (≤ 7 cm) has shifted significantly toward nephron-sparing surgery (NSS), given its equivalent oncological outcomes and superior functional preservation compared to radical nephrectomy (RN) [1]. However, the accuracy of clinical staging remains a persistent challenge. Despite advancements in cross-sectional imaging, approximately 10% to 12% of cT1 tumors are upstaged to pathological T3a (pT3a) postoperatively, typically due to microscopic invasion of the perirenal fat, renal sinus fat, or venous structures [2–4]. Pathological upstaging is not merely a staging discrepancy; it carries significant prognostic weight. Patients with upstaged disease face a higher risk of recurrence and decreased cancer-specific survival compared to those with pT1 disease [5–7]. For the clinician, the risk of upstaging influences critical surgical decisions, including the choice between NSS and RN, the breadth of the surgical margin, and the intensity of postoperative surveillance protocols. Several factors have been associated with upstaging to pT3a, including male sex, diabetes, higher BMI, tumor size, increased age, and hilar tumor location [3, 8, 9].

To address this preoperative uncertainty, several predictive models and nomograms have been developed. Notable among these are the models proposed by de la Barra et al. (2020) which emphasizes anatomical contact with main vessels [10]; Nocera et al. (2022), which incorporates comprehensive nephrometry and patient factors [11]; and the more recent model by Mei et al. (2024), which integrates morphological imaging features such as tumor margin irregularity and CT-based necrosis [12]. While these nomograms showed promising discriminatory power in their respective development cohorts, their performance often diminishes when applied to independent, diverse patient populations. Furthermore, comparisons of these specific models in a single external cohort are currently lacking in the literature. External validation is a prerequisite for the widespread clinical adoption of any predictive tool. The objective of this study was to perform external validation and to compare the Barra, Nocera, and Mei nomograms. By evaluating their discrimination, calibration, and clinical utility in a retrospective institutional cohort, we aim to identify the most reliable tool for predicting pT3a disease in patients presenting with cT1 renal masses.

## Materials and methods

### Study design

We conducted a retrospective study of patients who underwent NSS or RN for a cT1 renal mass at our institution between 2014 and 2025. Patients were identified through a search of the institutional surgical database. Inclusion Criteria: Age ≥ 18 years; presence of a single cT1 renal mass on preoperative cross-sectional imaging (CT or MRI); and available pathological report. Exclusion Criteria: Presence of multiple or bilateral renal masses; evidence of nodal or distant metastasis at presentation; hereditary renal cancer syndromes; or incomplete preoperative imaging/laboratory data required for the three nomograms.

### Data collection and outcome definition

We extracted demographic and medical history from the medical records. Preoperative laboratory results needed for evaluating the specific nomograms were also extracted. Radiological features were assessed by a single urologist (Y.S.), who was blinded to the final pathology report. Predicted probabilities for each patient were calculated by strictly applying the original scoring systems described in the respective primary publications. Specifically, the Barra nomogram utilizes maximum tumor diameter and gross radiological contact with the main renal vessels. The Nocera nomogram incorporates patient age, maximum tumor diameter, polar tumor location, medial rim location, and exophytic rate. The Mei nomogram integrates patient age, symptomatic presentation, smoking history, tumor size, hilar tumor involvement, platelet-to-lymphocyte ratio (PLR), tumor contour irregularity, and CT-based necrosis.

The primary outcome was pathological upstaging, defined as pT3a disease in the pathology report. This encompasses microscopic invasion of the perirenal fat, renal sinus fat, or the renal vein and its segmental branches, as outlined by the American Joint Committee on Cancer (AJCC) 8th Edition TNM staging system.

### Statistical analysis

Categorical variables were described as frequencies and percentages. Continuous variables were reported as medians and interquartile ranges (IQR). Logistic regression was performed for each nomogram variable. Odds ratios (OR) and 95% confidence intervals (CI) were calculated.

All aspects of model validation, including the assessment of discrimination and calibration, were performed in accordance with the TRIPOD statement guidelines [13]. Model discrimination was assessed using the Area Under the Receiver Operating Characteristic (ROC) Curve (AUC). Overall predictive accuracy and "goodness-of-fit" were quantified using Nagelkerke’s R^2^ and the Brier Score. Calibration was evaluated using parametric and nonparametric methods. The slope and intercept were calculated based on parametric calibration, along with the unreliability Index (U) and its associated p-value, which were used to test the null hypothesis of perfect reliability. Flexible Loess-smoothed curves were generated to visualize "calibration drift" across the risk spectrum. We calculated the Maximum Calibration Error (E_max_), the average error (E_avg_), and the 90th percentile error (E_90_) to identify localized model instability.

To assess the clinical relevance of the models, Standardized Decision Curve Analysis (sDCA) was performed [14, 15]. We calculated the Standardized Net Benefit (sNB) across a range of threshold probabilities to determine the clinical utility of each nomogram relative to default strategies ("assume all upstaged" vs. "assume none upstaged"). The 15% threshold was pre-specified as the most clinically relevant for surgical decision-making between NSS and RN. Statistical analysis was performed using R version 4.5.2 with the pROC, rms, and rmda packages. Two-sided *p* < 0.05 was considered statistically significant.

### Ethical considerations

The study was conducted in accordance with the ethical principles of the Declaration of Helsinki. Approval for the study was obtained from the Institutional Review Board (Protocol 0194–25-MMC). Due to the retrospective nature of the study and the use of de-identified clinical data, the ethics committee waived the requirement for written informed consent. All patient data were accessed and handled in strict confidence to ensure privacy and were processed in compliance with national data protection regulations.

## Results

### Patient characteristics and pathological outcomes

A total of 111 patients (74 males, 37 females) participated in the study. The median age was 65 years (IQR 55–71), and the median tumor size was 3.5 cm (IQR 2.5–5.0). The tumor was in the right kidney in 54 cases (48.6%) and in the left kidney in 57 cases (51.4%). RN was performed in 19 cases (17.1%), while NSS was performed in 92 cases (82.9%). The most common histology was clear cell RCC in both groups. Pathological upstaging to pT3a was observed in 19 cases (17.1%). Surgical margins were positive in 16 cases, with 9 in the pT1-2 group and 7 in the pT3 group. Detailed clinical and pathological characteristics are summarized in Table 1.

**Table 1:** Patient’s baseline characteristics

	pT1-2 N = 92	pT3a N = 19
Age, years	63.5 (53.8–71.0)	68.0 (61.5–72.0)
Gender		
- Female	30 (32.6%)	7 (36.8%)
- Male	62 (67.4%)	12 (63.2%)
Diabetes	28 (30.4%)	11 (57.9%)
Hypertension	52 (56.5%)	11 (57.9%)
BMI, Kg/m^2^	27.7 (24.6–31.1)	27.7 (26.4–32.8)
Smoking	47 (51.1%)	10 (52.6%)
Symptoms	29 (31.5%)	9 (47.4%)
Tumor size, cm	3.3 (2.5–4.5)	5 (3.8–6.1)
Tumor side		
- Right	44 (47.8%)	10 (52.6%)
- Left	48 (52.2%)	9 (47.4%)
Positive surgical margins	9 (9.8%)	7 (36.8%)
Tumor histology		
- clear cell	74 (80.5%)	15 (78.9%)
- papillary	12 (13.0%)	3 (15.8%)
- chromophobe	6 (6.5%)	1 (5.3%)

*BMI* body-mass index

### Univariate regression analysis of known risk factors

To validate the established predictors within our cohort, univariate logistic regression was performed on individual clinical and radiological variables (Table 2). Among demographic characteristics, age had an OR of 1.01 (95% CI 1.00–1.01, *p* = 0.067). Tumor size was a significant predictor of upstaging (OR 1.05; 95% CI 1.01–1.10; *p* = 0.012). Among radiological features, touching the main vessels was a significant predictor of pT3 (OR 1.37, 95% CI 1.16–1.63, *p* < 0.001). Polar involvement had an OR of 1.13 (95% CI 0.98–1.30, *p* = 0.087).

**Table 2:** Univariable regression analysis

Variable	Nomogram^a^	pT1-2 N = 92	pT3a N = 19	OR (95% CI)	*p*
Demographics					
Age, years	BNM	63.5 (53.8–71.0)	68.0 (61.5–72.0)	1.01 (1.00–1.01)	0.067
Symptoms	M	29 (32%)	9 (48%)	1.11 (0.95–1.28)	0.188
Smoking	M	47 (51%)	10 (53%)	1.01 (0.88–1.16)	0.903
Imaging					
Tumor size, cm	NM	3.3 (2.5–4.5)	5 (3.8–6.1)	1.05 (1.01–1.10)	0.012
Clinical stage, cT1b	B	34 (37%)	13 (68%)	1.20 (1.01–1.10)	0.011
Touches main vessels	B	12 (13%)	9 (47%)	1.37 (1.16–1.63)	< 0.001
Rim location, medial	N	36 (39%)	7 (37%)	0.99 (0.85–1.14)	0.854
Exophytic Rate	N				
- ≥ 50%		42 (46%)	7 (37%)	Ref	Ref
- < 50%		38 (41%)	7 (37%)	1.01 (0.87–1.18)	0.871
- Endophytic		12 (13%)	5 (26%)	1.16 (0.94–1.43)	0.158
Polar involvement	N	43 (47%)	13 (69%)	1.13 (0.98–1.30)	0.087
Hilus involvement	M	9 (10%)	4 (21%)	1.17 (0.94–1.45)	0.167
Necrosis	M	55 (60%)	15 (79%)	1.12 (0.97–1.30)	0.117
Edge irregularity	M	45 (49%)	13 (68%)	1.12 (0.97–1.29)	0.123
Laboratory					
PLR	M	121 (96–158)	117 (102–174)	1.00 (1.00–1.00)	0.670

^a^Included in the specified nomogram(s): B, Barra; N, Nocera; M, Mei

*CI* confidence interval, *OR* odds ratio, *PLR* platelet-to-lymphocyte rate

### Performance of the nomograms: discrimination

**Fig. 1:**
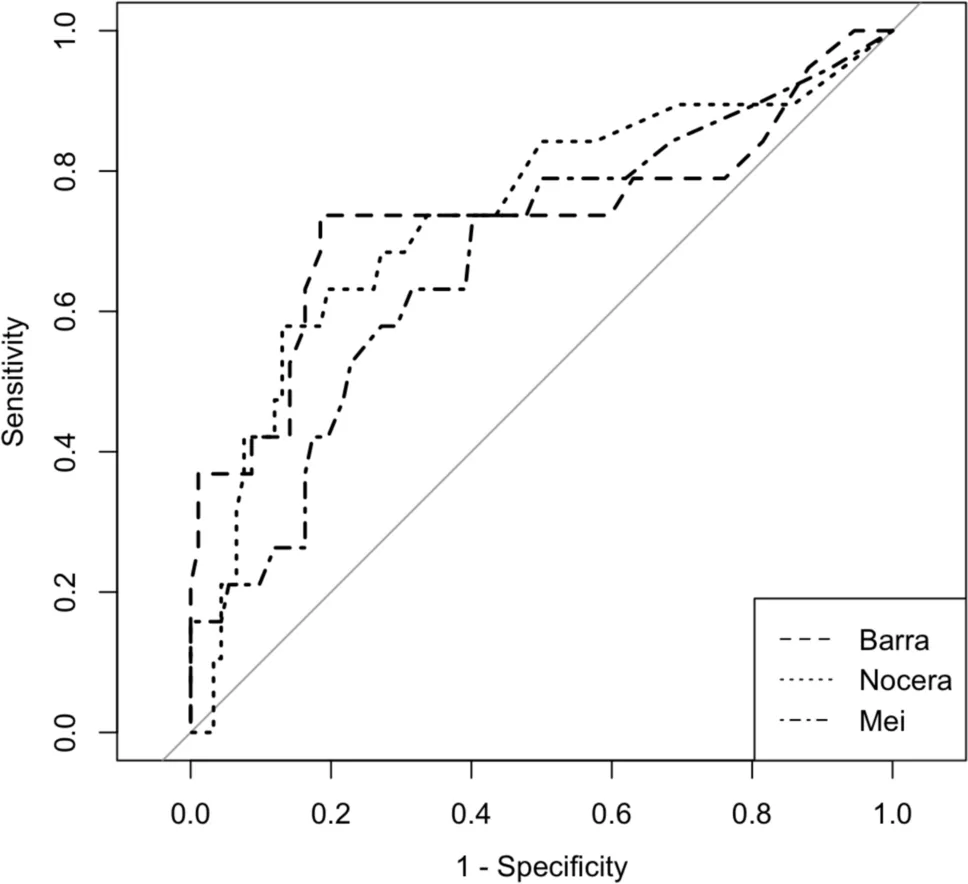
Receiver operating characteristic curves. The AUC is 0.733 (95% CI 0.576–0.889), 0.736 (95% CI 0.597–0.875), and 0.677 (95% CI 0.536–0.819) for the Barra, Nocera, and Mei nomograms, respectively. No statistically significant difference between the AUCs is observed in the DeLong test (*p* > 0.05 for all pairs). *AUC* area under the curve; *CI* confidence interval

Model discrimination was evaluated through ROC analysis (Fig. 1). The Nocera and Barra nomograms demonstrated the highest accuracy, with nearly identical AUC values of 0.736 (95% CI 0.597–0.875) and 0.733 (95% CI 0.576–0.889), respectively. Both outperformed the Mei nomogram, which had an AUC of 0.677 (95% CI 0.536–0.819). The DeLong test showed no statistically significant differences in AUCs (*p* > 0.05 for all pairs).

### Global calibration and reliability (parametric)

**Fig. 2:**
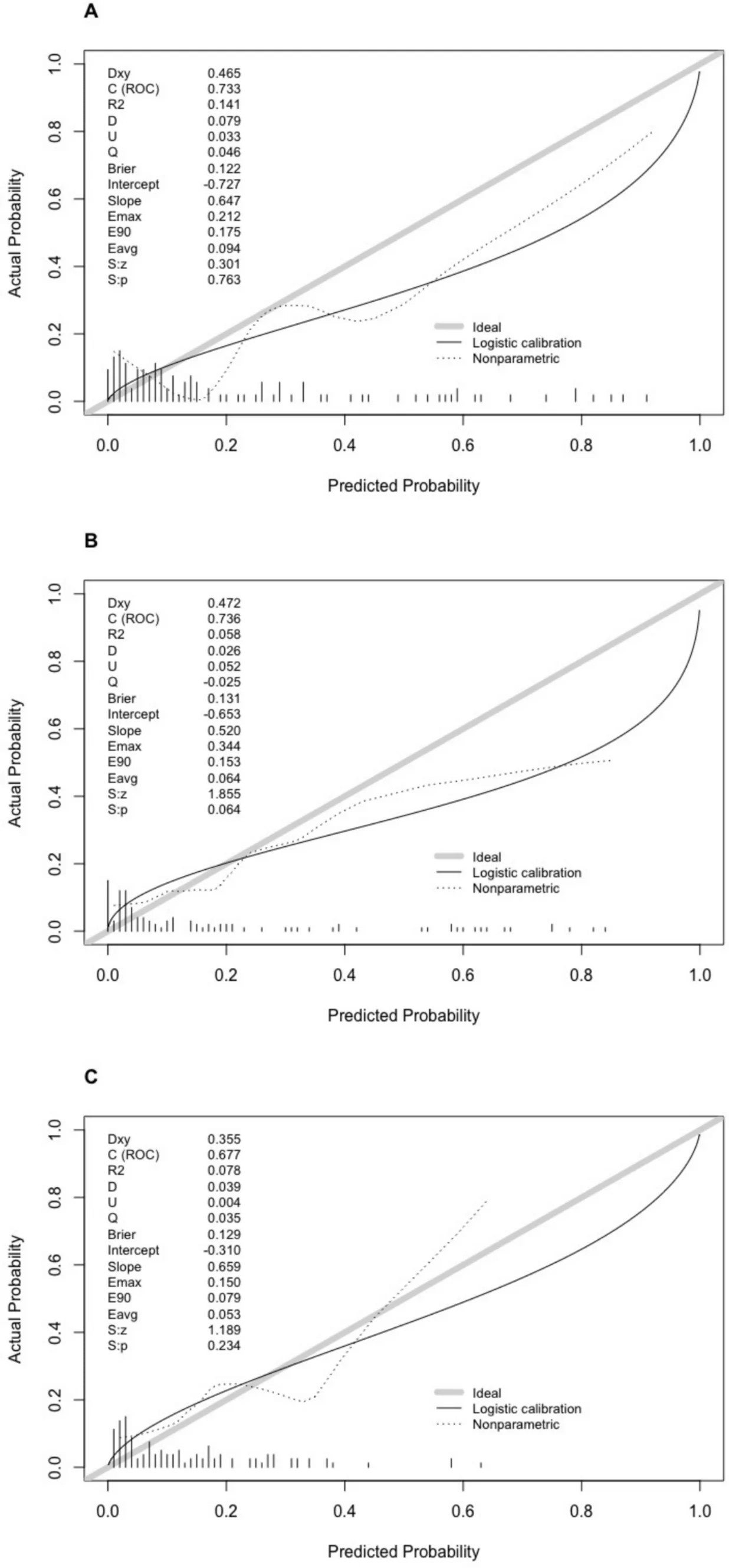
Calibration plots. Logistic (parametric) calibration (solid line) and non-parametric calibration (dashed line) of (A) Barra, (B) Nocera, and (C) Mei nomograms. All models showed negative intercepts and calibration slopes below 1. The Mei nomogram demonstrated the lowest Unreliability index (U = 0.004, *p* = 0.294). Nocera nomogram showed significant evidence of unreliability (U = 0.052, *p* = 0.021). The Barra nomogram indicated borderline reliability (U = 0.033, *p* = 0.058). The Mei nomogram demonstrated the greatest stability across the risk spectrum, with the lowest maximum calibration error (E_max_ = 0.150). The Barra nomogram showed numerically higher predictive performance, with the lowest Brier Score (0.122) and the highest explained variance (Nagelkerke R^2^ = 0.141)

All three models showed negative intercepts (ranging from -0.310 to -0.727) and calibration slopes below 1 (ranging from 0.520 to 0.659), indicating a general tendency to overestimate the risk of upstaging in this cohort (Fig. 2). However, reflecting the limited statistical power of the cohort, the 95% CI for certain model slopes encompassed 1.0, and specific intercepts encompassed 0.0, indicating that these calibration distortions did not always reach statistical significance (Table 3). A comprehensive calibration analysis revealed notable differences in model reliability. Despite its lower discrimination, the Mei nomogram proved to be the most statistically reliable tool in our population. It demonstrated the lowest Unreliability index (U = 0.004) and was the only model with a non-significant p-value (*p* = 0.294), indicating that its predicted probabilities were consistent with the observed outcomes. Conversely, the Nocera nomogram showed significant evidence of unreliability (U = 0.052, *p* = 0.021), suggesting a statistically significant mismatch between predicted risks and actual pathology. The Barra nomogram indicated borderline reliability (U = 0.033, *p* = 0.058).

**Table 3:** Comparative nomogram performance metrics

Metric (95% CI)	Barra	Nocera	Mei
AUC (C-statistic)	0.733 (0.576–0.889)	0.736 (0.597–0.875	0.677 (0.536–0.819
Calibration Slope	0.647 (0.29–1.17)	0.520 (0.21–0.96)	0.659 (0.14–1.25)
Calibration Intercept	-0.727 (-1.43-[-0.16])	-0.653 (-1.44–0.11)	-0.310 (-1.43–0.69)
Nagelkerke R^2^	0.141 (-0.27–0.42)	0.058 (-0.44–0.39)	0.078 (-0.21–0.29)
Unreliability (U / p)	0.033 (-0.01–0.15, p = 0.058)	0.052 (-0.01–0.22, p = 0.021)	0.004 (-0.01–0.12, p = 0.294)
Brier Score	0.122 (0.08–0.16)	0.131 (0.09–0.18)	0.129 (0.08–0.18)
Resolution (Q)	0.046 (-0.29–0.29)	-0.025 (-0.44–0.27)	0.035 (-0.25–0.18)
Max Error (E_max_)	0.212 (015–0.49)	0.344 (0.11–0.67)	0.150 (0.11–0.39)
Average Error (E_avg_)	0.094 (0.06–0.15)	0.064 (0.03–0.13)	0.053 (0.03–0.11)
90th percentile Error (E_90_)	0.175 (0.12–0.36)	0.153 (0.07–0.37)	0.079 (0.06–0.22)

*AUC* area under the curve, *CI* confidence interval

### Nonparametric calibration (flexible loess smoothing)

To identify specific failure risk thresholds, we performed a nonparametric calibration analysis using Loess-smoothed flexible curves (Fig. 2). The Mei nomogram proved to be the most stable and precise tool for patient counseling; its absolute risk predictions closely tracked actual pathology across nearly the entire cohort, yielding the smallest maximum error (E_max_ = 0.150). In contrast, while the Barra and Nocera models are good at ranking patients from low to high risk, they suffer from severe "calibration drift" when a patient’s calculated risk is high. At these upper risk thresholds, both models consistently and significantly overestimate the true likelihood of pathological upstaging. The Nocera nomogram was particularly unstable in this high-risk zone, showing a massive worst-case error margin, with predictions drifting from actual outcomes by over 34% (Table 3).

### Overall predictive accuracy and resolution

The Barra nomogram showed numerically higher predictive performance, with the lowest Brier Score (0.122) and the highest explained variance (Nagelkerke R^2^ = 0.141). Additionally, Barra had the highest resolution (Q = 0.046), indicating it provides the clearest risk separation for clinical decision-making. However, consistent with the constraints of our overall cohort size, the numeric variations among the three models were minor, and the differences in overall predictive accuracy did not reach statistical significance (Table 3).

### Clinical utility

**Fig. 3:**
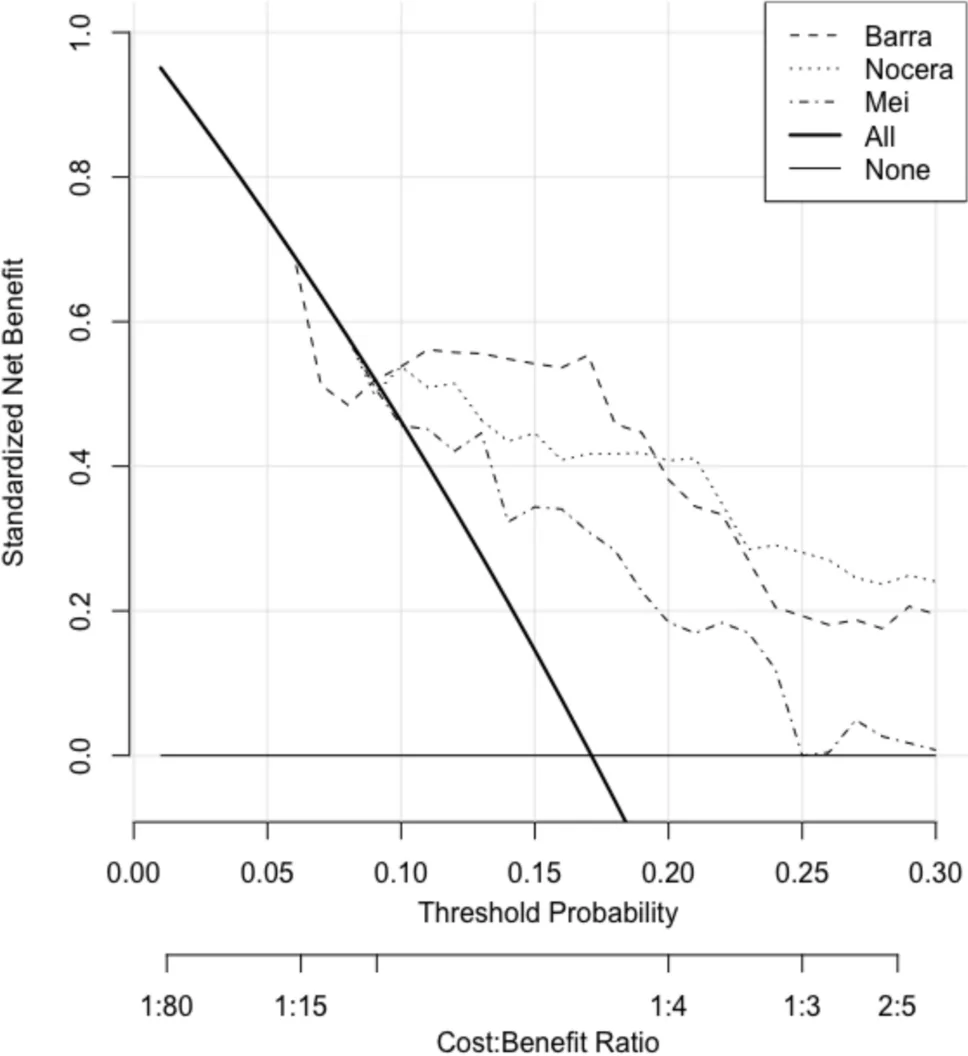
Decision curve analysis. The Barra nomogram achieved the highest sNB within the 8% to 18% risk threshold range, capturing about 55% of the maximum potential benefit at the 15% risk threshold. In the higher-risk range (> 18%), the Nocera nomogram provided the highest sNB. *sNB* standardized net benefit

The clinical utility of the three nomograms was further evaluated using sNB to account for the institutional upstaging rate (Fig. 3). All three models showed positive sNB for threshold probabilities above 8%, indicating greater clinical value than default strategies. The Barra nomogram achieved numerically higher sNB within the relevant 8% to 18% risk threshold range, capturing about 55% of the maximum potential benefit at the 15% risk threshold. In the higher-risk range (> 18%), the Nocera nomogram provided the highest sNB. The Mei nomogram, while still maintaining a positive sNB, consistently offered lower standardized utility than the Barra and Nocera models.

## Discussion

In this study, we externally validate and compare the performance of the Barra, Nocera, and Mei nomograms in our cohort [10–12]. Regression analysis confirms known risk factors, such as tumor size and proximity to main vessels, as predictors of upstaging. This validates our cohort for the validation process.

In this independent external validation, all three nomograms exhibited moderate discriminative ability, with AUCs ranging from 0.677 to 0.736. These values are lower than those reported in the original development studies (usually above 0.80), a pattern common in external validations due to variations in institutional case mix. Specifically, the Mei nomogram showed the lowest discriminatory power (AUC = 0.677), but the differences were not statistically significant. This may suggest that the Mei model is less effective at distinguishing between patients at low and high risk of pathological upstaging in our population, whereas the Nocera and Barra models maintain a stronger ability to rank patients by upstaging risk. Notably, the Nocera nomogram has previously been externally validated in a series of 288 patients, with a similar AUC (0.766) [16].

All nomograms exhibited a consistent pattern of over-optimism. Coupled with the negative intercepts observed across all models, these findings may suggest a general overestimation of the risk of pathological upstaging in our cohort. While mathematical recalibration (adjusting the calibration intercept and slope) is frequently utilized to align predictive models with local prevalence, we explicitly chose not to perform it. Given our limited number of upstaging events, attempting a formal logistic recalibration would incur a high risk of model overfitting and statistical instability.

sDCA showed that all three nomograms offered a positive net benefit for threshold probabilities above 8%, outperforming the default strategies of "assume all upstaged" or "assume none upstaged." The Barra nomogram demonstrated the highest clinical utility in the decisive 8%-18% risk range, the critical window for choosing between RN and NSS. Nocera provided a greater net benefit at higher threshold probabilities, while Mei consistently delivered lower standardized utility across the clinical spectrum. In addition to having the lowest Brier Score (0.122), the Barra model provides the most robust clinical benefit for routine surgical decisions in this cohort.

From a clinical perspective, the role of preoperative prediction of pathological upstaging remains nuanced. While an increased estimated risk may influence surgical planning, it should not be the sole determinant in choosing between NSS and RN. Surgical decisions are inherently multifactorial and must incorporate patient-specific factors such as baseline renal function, comorbidities, oncological risk tolerance, and patient preference. These models may have value beyond guiding the choice of surgical approach. Preoperative risk stratification could help identify patients for whom active surveillance may be less appropriate, particularly when the estimated risk of adverse pathological features is elevated. In addition, such estimates may support patient counseling and help tailor the intensity of postoperative surveillance.

A key insight from our data is the gap between discrimination and calibration. The Nocera nomogram exemplifies this paradox; it showed an AUC of 0.736 but performed worst at accurately reflecting absolute risk. Its high E_max_ indicates that, at certain risk thresholds, the model’s predictions can differ from observed outcomes by more than 34%. In contrast, the Mei nomogram, despite its lower AUC (0.677), demonstrated the greatest consistency across the risk spectrum (E_max_ = 0.150). For a surgeon, this suggests that the Mei model provides a more dependable tool for absolute risk counseling, while the Nocera model may be more appropriate for relative risk stratification, where the patient’s rank within the cohort is more important than the exact percentage.

Standard metrics often miss real-world surgical impacts. Our sDCA offers essential clinical evidence. Within the 8% to 18% threshold, the Barra nomogram has the highest sNB. The Barra model provides a good balance of performance metrics, capturing more clinical benefit by balancing Nocera’s high discrimination and Mei’s reliable calibration.

Recent evidence shows the complexity of decision-making for suspected pT3a disease. A recent propensity score-matched analysis suggests NSS may have oncologic outcomes similar to RN in selected pT3a patients, challenging the idea that higher preoperative risk should lead to more aggressive surgery [17]. Modern risk frameworks, including KEYNOTE-564 and guideline-based systems, stress the importance of postoperative pathological and biological risk assessments over preoperative estimates [18]. These findings highlight the need to interpret preoperative nomograms cautiously, using them mainly for risk stratification rather than definitive treatment, especially as multimodal and biology-driven strategies evolve.

Our cohort’s upstaging rate (17.1%) exceeds that of original cohorts. It reflects a real-world surgical population, including all eligible patients without filters, which enhances applicability but adds heterogeneity due to differences in baseline risk, imaging, and surgical criteria. These factors may cause calibration drift and overestimation of risk by all models when applied to higher-prevalence populations. Local validation and recalibration are essential before using these predictions for surgical decisions.

This study’s limitations include its retrospective, single-center design, small sample size, few upstaging events, and long study period, which may be affected by changes in imaging protocols over time. These factors may weaken model validation and risk overinterpreting performance differences. While representative of current practice, the limited upstaging events restrict definitive model comparisons. Radiological features were assessed by one urologist, ensuring consistency but ignoring inter-observer variability across institutions. Future work should explore simple recalibration to improve model accuracy. Predictive nomograms must be viewed amid evolving diagnostics, as advances in imaging, biopsy, and biomarkers could lessen reliance on static tools. Thus, nomograms should serve as adjuncts, not standalone decisions. Our results show performance differences and highlight the limits of fixed models on diverse populations.

We recognize that a limitation of this study is the histological mismatch in our validation. The Nocera nomogram was developed only for clear cell RCC, but we applied it to a mixed cohort that included papillary and chromophobe types. Preoperative biopsies are usually not performed for most cT1 masses, so final tumor histology is unknown during surgical planning, making evaluation of these nomograms in a mixed cohort more reflective of real-world use. However, clinicians should note that applying clear cell-specific models to mixed populations may cause calibration issues. Furthermore, due to the heterogeneity of renal cell carcinoma and differences in tumor biology across histological subtypes, broad predictive models have limited applicability. This highlights the need for more individualized, biology-driven risk assessment tools. Given these limitations and the resulting statistical uncertainty, the comparative findings of this study should be considered exploratory and hypothesis-generating rather than definitive.

## Conclusion

The Barra, Nocera, and Mei nomograms are useful tools for identifying cT1 renal masses at high risk of upstaging to pT3. All nomograms performed lower than originally reported. For active surgical planning, the Barra nomogram appeared to provide a favorable balance of performance in this cohort, providing better clinical utility within the relevant probability range. Since all models tend to overestimate risk at higher thresholds, surgeons should exercise caution when advising patients at the upper end of the risk spectrum.

## Data Availability

No datasets were generated or analysed during the current study.

## Declarations

### Declaration of AI and AI-assisted technologies in the writing process

During the preparation of this work, the authors used Gemini 3.1 to improve language, rephrasing, grammar, and spelling. After using this tool, the authors reviewed and edited the work and take full responsibility for the content of the publication.
